# Targeting Interleukin-6/Glycoprotein-130 Signaling by Raloxifene or SC144 Enhances Paclitaxel Efficacy in Pancreatic Cancer

**DOI:** 10.3390/cancers15020456

**Published:** 2023-01-11

**Authors:** Nina A. Hering, Emily Günzler, Marco Arndt, Miriam Zibell, Johannes C. Lauscher, Martin E. Kreis, Katharina Beyer, Hendrik Seeliger, Ioannis Pozios

**Affiliations:** 1 Department of General and Visceral Surgery, Charité—Universitätsmedizin Berlin, Corporate Member of Freie Universität Berlin and Humboldt-Universität zu Berlin, 12203 Berlin, Germany; 2 IU Health University, 55116 Mainz, Germany

**Keywords:** pancreatic ductal adenocarcinoma, interleukin-6, paclitaxel, raloxifene, SC144, drug synergy, gp130, IL-6, STAT3, pancreatic cancer

## Abstract

**Simple Summary:**

Pancreatic cancer is presently the fourth most common cause of cancer-related death. Treatment options and prognosis are poor. Therefore, new therapeutic approaches are urgently needed. Molecular targeting of the interleukin-6/glycoprotein-130/signal transducer and activator of transcription 3 signaling cascade is a promising approach in pancreatic cancer therapy. The present study investigates the combined effect of the first-line chemotherapeutic paclitaxel with the small-molecule gp130 inhibitor SC144 and the non-steroidal selective estrogen receptor modulator raloxifene that both interfere with interleukin-6/glycoprotein-130 signaling. Experiments performed on a mouse model of pancreatic cancer and cell lines proved both molecules to enhance low-dose paclitaxel effects by increasing apoptosis in tumor cells or reducing interleukin-6 levels, respectively. These findings might be promising to improve treatment efforts in pancreatic cancer, while the paclitaxel side-effect profile could be improved by lowering paclitaxel doses.

**Abstract:**

Interleukine-6 plays a key role in the progression and poor survival in pancreatic ductal adenocarcinoma (PDAC). The present study aimed to clarify if targeting the interleukin-6/glycoprotein-130 signaling cascade using the small-molecule gp130 inhibitor SC144 or raloxifene, a non-steroidal selective estrogen receptor modulator, enhances paclitaxel efficacy. MTT/BrdU assays or TUNEL staining were performed to investigate cell viability, proliferation and apoptosis induction in L3.6pl and AsPC-1 human pancreatic cell lines. In vivo, effects were studied in an orthotopic PDAC mouse model. Tumor specimens were analyzed by qPCR, immunohistochemistry and ELISA. Combination of paclitaxel/raloxifene, but not paclitaxel/SC144, enhanced proliferation and viability inhibition and increased apoptosis compared to single treatment in vitro. Synergy score calculations confirmed an additive influence of raloxifene on paclitaxel. In the PDAC mouse model, both combinations of raloxifene/paclitaxel and SC144/paclitaxel reduced tumor weight and volume compared to single-agent therapy or control. Raloxifene/paclitaxel treatment decreased survivin mRNA expression and showed tendencies of increased caspase-3 staining in primary tumors. SC144/paclitaxel reduced interleukin-6 levels in mice’s tumors and plasma. In conclusion, raloxifene or SC144 can enhance the anti-tumorigenic effects of paclitaxel, suggesting that paclitaxel doses might also be reduced in combined chemotherapy to lessen paclitaxel side effects.

## 1. Introduction

Pancreatic ductal adenocarcinoma (PDAC) has a poor prognosis and is one of the most common causes of cancer-related death. This is correlated to its rapid progression, fast metastatic spread to the liver and spleen and resistance to chemotherapy [1]. Within recent years, the cytokine interleukine-6 (IL-6) was recognized to promote the development and progression of pancreatic cancer. IL-6 is elevated in the serum of pancreatic cancer patients and is assumed to play a key role in cachexia, advanced tumor stage and poor survival [2,3,4]. It directly affects the tumor cells, contributes to the generation of a pro-tumorigenic microenvironment [5], might be involved in angiogenesis and is an independent risk factor for hepatic metastasis in PDAC patients [6]. For signaling via the IL-6 receptor, which exists in a membrane-bound and soluble form, the ubiquitously expressed co-receptor glycoprotein-130 (gp130) is needed [7,8]. IL-6 binding results in the phosphorylation of the Janus kinases (JAK1, JAK2, TYK2) and subsequent activation of signal transducers and activators of transcription-1 and -3 (STAT1 and STAT3). Especially, the activation of STAT3 was shown to be linked to tumor growth, survival, angiogenesis and metastatic processes [9]. Aside from IL-6, gp130/STAT3 signaling can be activated by several other cytokines of the IL-6 family, including leukemia inhibitor factor, oncostatin M (OSM), ciliary neurotrophic factor, cardiotrophin-1, cardiotrophin-like cytokine, neuropoietin, IL-11, IL-27 and IL-31 [10]. Consequently, targeting the gp130/STAT3 signaling cascade seems to be a promising tool to inhibit tumorigenesis in PDAC. In previous studies, we already assessed two different molecules: the small inhibitor molecule SC144 [11] and the non-steroidal selective estrogen receptor modulator raloxifene [12]. Both molecules targeted the co-receptor gp130, inhibited IL-6- or OSM-stimulated STAT3 phosphorylation and had anti-proliferative effects in PDAC cell lines in vitro [11,12].

This gives rise to the question of how far raloxifene or SC144 could enhance or add to the anti-tumorigenic effects of standard chemotherapeutics. Presently, paclitaxel-gemcitabine is an effective first-line chemotherapeutic treatment option in advanced PDAC. However, patients frequently suffer from diverse side effects, including fatigue, nausea, vomiting, diarrhea, neurotoxicity, mucositis, neutropenia or thrombocytopenia [13]. Raloxifene is already approved for preventing and treating osteoporosis and has a better drug compatibility profile [14] compared to paclitaxel [13]. SC144 is not clinically approved so far and needs to be studied in detail for a safety evaluation.

With respect to improving therapy efforts and reducing chemotherapy dosage, including its associated side effects, the present study investigates PDAC cell lines and a PDAC mouse model to clarify if the gp130/STAT3-interfering molecules raloxifene or SC144 enhance paclitaxel efficacy.

## 2. Material and Methods

### 2.1. Cell Culture and Reagents

The human PDAC cell line L3.6pl is a secondary, highly metastatic human pancreatic adenocarcinoma cell line derived from an orthotopic mouse xenograft model [15]. AsPC-1 cells (ASPC) were obtained from the American Type Culture Collection (Rockville, MD, USA). Cells were cultured using Dulbecco’s Minimal Essential Medium (DMEM, Thermo Fisher Scientific, Darmstadt, Germany), supplemented with 10% fetal bovine serum and 1% penicillin-streptomycin (Thermo Fisher Scientific) in a humidified atmosphere of 5% CO_2_ at 37 °C.

For in vitro experiments, raloxifene, the quinoxalinhydrazide derivative SC144 and paclitaxel (all Sigma-Aldrich; Schnelldorf, Germany) were dissolved in 0.1% dimethylsulfoxide (DMSO, Carl Roth GmbH & Co. KG, Karlsruhe, Germany). For in vivo studies, all substances were dissolved in 10% DMSO and corn oil (Sigma-Aldrich).

### 2.2. Proliferation and Viability Assays

L3.6pl or ASPC cells were seeded in 96-well microplates (5000 cells/well). After growing overnight at 37 °C, the cells were shifted to serum-free conditions and were challenged in sextuplicate with raloxifene (1, 5, or 10 µM), SC144 (0.1, 0.2, 1, or 2 µM) and paclitaxel (1, 5 or 10 nM) alone or in combination for 48 h. Cell proliferation was assessed by 5-bromo-2′-deoxyuridine (BrdU) incorporation assay (Roche, Mannheim, Germany), and cell viability was determined using 3-[4,5-dimethylthiazol-2-yl]-2,5-diphenyltetrazolium bromide (MTT) assay (Sigma-Aldrich). The assays were run accordingly to the manufacturers’ protocols.

### 2.3. Synergism Calculation

Synergy scores of raloxifene/paclitaxel or SC144/paclitaxel were calculated by the web application SynergyFinder https://synergyfinder.fimm.fi (accessed on 9 June 2022), which can be used for interactive analysis and visualization of synergistic drug combinations in pre-clinical model systems [16]. The following combinations were assessed in MTT and BrdU assays, and the results were uploaded to Synergy Finder: raloxifene (0, 1, 5, 10 µM), SC144 (0, 1, 5, 10, 20 µM) and paclitaxel (0, 1, 5, 10 nM). For the calculation of synergy scores, the zero interaction potency (ZIP) model [17] was used. For synergy scores less than -10, the drug interaction is assumed to be antagonistic. At values between -10 to 10, the drugs are likely to act additive, and at a synergy score of more than 10, the interaction is expected to be synergistic (https://synergyfinder.fimm.fi).

### 2.4. TUNEL Staining

L3 6pl cells were challenged with raloxifene (10 µM), SC144 (10 µM) and/or paclitaxel (10 nM; 100 nM) for 48 h. Apoptosis was determined by TdT-mediated dUTP-biotin nick end labeling (TUNEL; In-situ Cell Death Detection Kit-Fluorescein, Sigma-Aldrich) according to the manufacturer’s instructions. Labeled DNA strand breaks in apoptotic cells were detected and quantified by fluorescence-activated cell sorting. Raloxifene or SC144 was evaluated as a single agent or in combination with paclitaxel. Staurosporine (1 µM) served as the positive control; negative controls were performed without TUNEL staining.

### 2.5. PDAC Mouse Model

Male BALB/c nu/nu mice were purchased from Janvier (Le Genest-Saint-Isle, France). The animals were kept under a 12 h/12 h day/night cycle with free access to food and water. Experiments were performed with eight weeks old mice with a body weight of 20–25 g. All animal experiments were approved by the regional authority (Landesamt für Gesundheit und Soziales, Berlin, G0212/17) and were performed in compliance with the European Union guideline 2010/63/EU.

Anesthesia was done with ketamine (120 mg/kg body weight) and xylazin (8 mg/kg body weight) (both Pfizer, Berlin, Germany) by intraperitoneal injection. Analgesia was achieved by subcutaneous carprofene (5 mg/kg body weight) injection. Laparotomy and pancreas mobilization was carried out as described earlier [15,18]. A total of 10^6^ L3.6pl cells in 10 µL medium (DMEM) were injected into the pancreatic tail, the pancreas was repositioned, and the abdomen was closed with a suture.

Treatment was performed by intraperitoneal injection and started two days after orthotopic injection. The mice were randomly assigned to six different treatment groups: control (vehicle solution at the same volume), raloxifene (30 mg/kg body weight), SC144 (100 mg/kg body weight), paclitaxel (10 mg/kg body weight), raloxifene/paclitaxel (30 mg/kg body weight raloxifene plus 10 mg/kg body weight paclitaxel) and SC144/paclitaxel (100 mg/kg body weight SC144 plus 10 mg/kg body weight paclitaxel). Each treatment group was performed by five animals, and each experiment was run at least two times independently. Vehicle control, raloxifene and SC144 were injected every day, and paclitaxel every four days. General condition, weight loss and abundant tumor growth were monitored every day. After 26 days of treatment, all mice were anesthetized, and blood was collected by aspirating blood from the heart. Serum was stored at −80 °C. After sacrifice, the metastatic spread was assessed, primary tumors were removed and weight and size were determined for calculating tumor volume (tumor volume = length × width × height). Tumor samples were frozen in liquid nitrogen or fixated in formalin for further investigations.

### 2.6. Quantitative Realtime-RT-PCR

RNA was extracted from mouse tumors using NucleoSpin^®^ RNA (Macherey-Nagel, Düren, Germany). RT-PCR was performed with a High-Capacity cDNA Archive Kit (Thermo Fisher Scientific). Quantitative realtime-PCR was set up in duplicates and run in a Quant Studio 3 realtime-PCR cycler (Life Technologies GmbH, Darmstadt, Germany). The following TaqMan Gene Expression Assays (all Thermo Fisher Scientific) were used: IL-6 (Hs00174131_m1), survivin (Hs00153353_m1), MMP-7 (Hs01042796_m1), STAT3 (Hs00374280_m1) and 28 sRNA (Hs00396989_m1) as endogenous control. Relative gene expression was calculated from threshold cycle values after normalization to endogenous control using Quant Studio 3 software.

### 2.7. Caspase-3 Staining

Paraffin-embedded tumor sections were cut (4 µm), deparaffinized, rehydrated and heated in sodium citrate solution at pH 6.0 for epitope retrieval. 0.5% Triton X-100 was used for permeabilization and 1% bovine serum albumin and 5% goat serum for blocking. Primary antibody staining was carried out with anti-caspase-3 (cleaved) (Cell Signalling, #9661) at 4 °C overnight. This was followed by treatment with biotin-conjugated polyclonal swine anti-rabbit secondary antibody (E0353, Agilent Technologies, Santa Clara, CA, USA) and phosphatase-conjugated streptavidin (Seracare Life Sciences, Milford, MA, USA). For visualization, HistoMark^®^ RED (Seracare Life Sciences) and hematoxylin counterstaining (Sigma-Aldrich) were performed. Quantification was done with ImageJ (Fiji) freeware using the color threshold tool. Caspase-positive cells were counted per area.

### 2.8. Enzyme-Linked Immunosorbent Assay (ELISA)

Interleukin-6 concentrations in mouse plasma were determined using human or murine-specific IL-6 Quantikine ELISAs (both R&D Systems, Minneapolis, MN, USA) according to the manufacturer’s instructions.

### 2.9. Statistical Analysis

GraphPad PRISM statistics software (San Diego, CA, USA) was used for statistical analysis. Statistical significance was considered by one-way ANOVA comparison or Student’s *t*-test adjusted to Bonferroni-Holm. All values are given as mean ± SEM. Differences of *p* < 0.05 were considered significant (* *p* < 0.05; ** *p* < 0.01; *** *p* < 0.001).

## 3. Results

The potential of raloxifene or SC144 to enhance the inhibitory effect of paclitaxel on cell viability and proliferation was studied in MTT and BrdU assays in L3.6pl and ASPC cells. Based on the results obtained from MTT and BrdU assays in L3.6pl, dose-response matrices (supplementary Figures S1 and S2) and synergy scores were calculated to estimate the degree of drug interactions.

### 3.1. Raloxifene/Paclitaxel Act Synergistic or Additive to Reduce Cell Viability and Proliferation In Vitro

**Figure 1:**
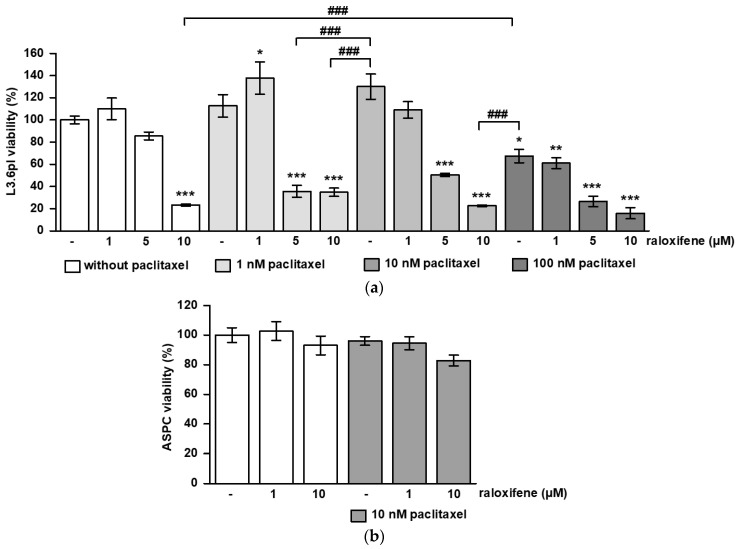

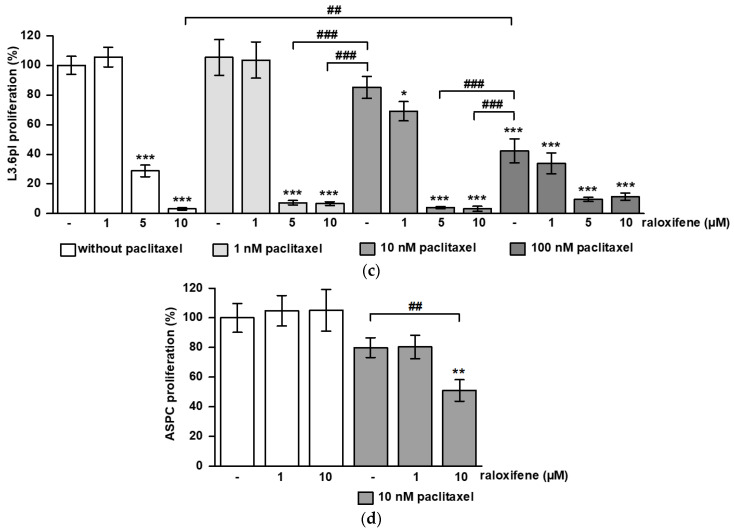
Synergistic and additive effects of raloxifene and paclitaxel on cell viability and proliferation. L3.6pl and ASPC cells were treated with different concentrations of raloxifene and paclitaxel alone or in combination. The graphs show the results obtained from MTT and BrdU assays on (**a**) cell viability in L3.6pl, (**b**) cell viability in ASPC, (**c**) proliferation in L3.6pl and (**d**) proliferation in ASPC. The data are given as mean values ± SEM from at least two independent experiments; the mean control value was set to 100%. * *p* < 0.05, ** *p* < 0.01 and *** *p* < 0.001 versus control; ^##^ *p* < 0.01 and ^###^ *p* < 0.001 versus paclitaxel.

In L3.6pl cells, 10 µM raloxifene and 100 nM paclitaxel alone reduced cell viability significantly compared to untreated controls (*p* < 0.001), at which 10 µM raloxifene was more effective than 100 nM paclitaxel (*p* < 0.001) (Figure 1a). While 1 and 10 nM paclitaxel alone were not sufficient to reduce viability, this was achieved by combined treatment with 5 or 10 µM raloxifene (*p* < 0.001) (Figure 1a). Most interestingly, a combined treatment with 10 µM raloxifene plus 10 nM paclitaxel was more efficient than a single treatment with 100 nM paclitaxel (*p* < 0.001) (Figure 1a). The synergy score calculated from these data was 11.4, pointing to a synergistic action of raloxifene and paclitaxel in reducing cell viability. ASPC cells turned out to be less sensitive, as 10 µM raloxifene plus 10 nM paclitaxel did not diminish cell viability significantly (Figure 1b). Single treatment with 5 µM, 10 µM raloxifene or 100 nM paclitaxel reduced proliferation compared to control in L3.6pl (*p* < 0.001) (Figure 1c). Combined treatment of 5 µM raloxifene or 10 µM raloxifene with 1 nM or 10 nM paclitaxel reduced proliferation compared to control (*p* < 0.001) and single paclitaxel treatment (*p* < 0.001) (Figure 1c). The 10 nM paclitaxel combined with 5 or 10 µM raloxifene was more efficient in proliferation inhibition than 100 nM paclitaxel alone (*p* < 0.001) (Figure 1c). Additionally, in ASPC cells, combined treatment with 10 µM raloxifene plus 10 nM paclitaxel was more effective than paclitaxel alone (*p* < 0.01 versus control and versus paclitaxel) (Figure 1d). The synergy score calculated was 7.5, suggesting an additive interaction of both drugs on proliferation.

### 3.2. SC144 Acts Antagonistic on Paclitaxel-Induced Viability and Proliferation Inhibition In Vitro

**Figure 2:**
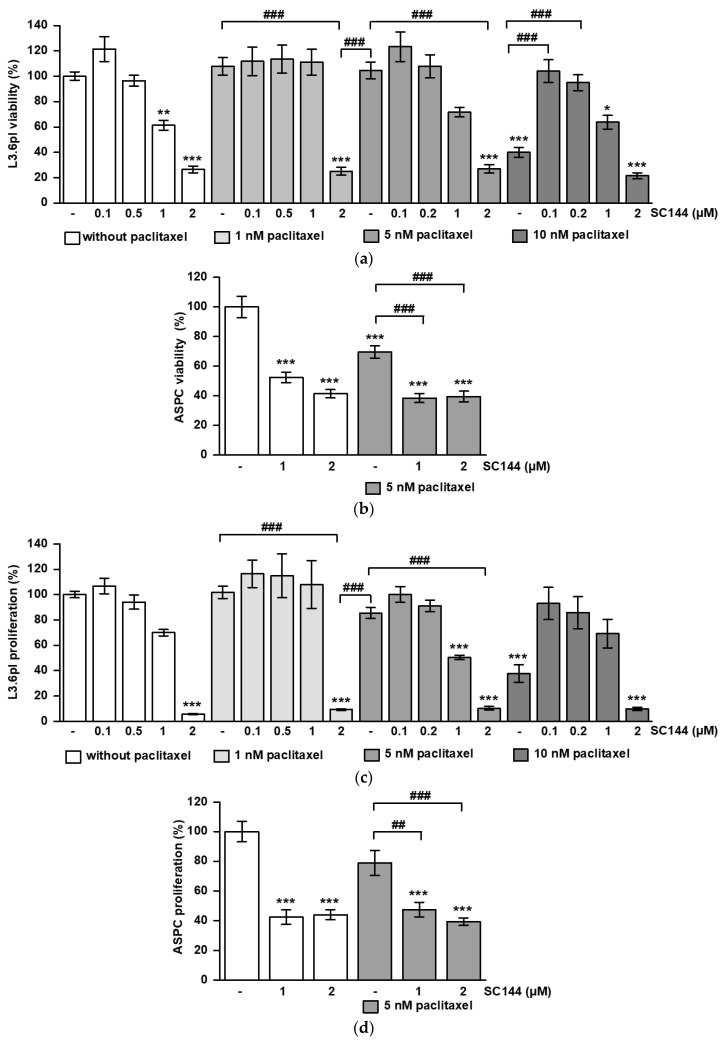
Effects of SC144 and paclitaxel on cell viability and proliferation. L3.6pl and ASPC cells were treated with different concentrations of SC144 and paclitaxel alone or in combination. The graphs show the results obtained from MTT and BrdU assays on (**a**) cell viability in L3.6pl, (**b**) cell viability in ASPC, (**c**) proliferation in L3.6pl, and (**d**) proliferation in ASPC. The data are given as mean values ± SEM from at least two independent experiments; the mean control value was set to 100%. * *p* < 0.05, ** *p* < 0.01 and *** *p* < 0.001 versus control; ^##^ *p* < 0.01 and ^###^ *p* < 0.001 versus paclitaxel.

The 1 and 2 µM SC144, as well as 10 nM paclitaxel alone, inhibited cell viability in L3.6pl cells (*p* < 0.01, *p* < 0.001) (Figure 2a). While 1 or 5 nM paclitaxel alone had no effect, combination with 2 µM SC144 diminished viability respectively (*p* < 0.001 versus control and versus single paclitaxel treatment). However, combined treatment with 0.1 µM (*p* < 0.001), 0.2 µM (*p* < 0.001) and 1 µM SC144 blocked the effect of 10 nM paclitaxel on viability reduction (Figure 2a). This is also reflected by the synergy score of -16, suggesting that SC144 and paclitaxel act antagonistic. In ASPC cells, a dose of 5 nM paclitaxel was tested that inhibited cell viability (*p* < 0.001) (Figure 2b). The 1 and 2 µM SC144 alone reduced cell viability as well (*p* < 0.001 versus control and versus paclitaxel). This effect was not further enhanced by paclitaxel in combination (Figure 2b). Very similar results were obtained from proliferation assays. The 2 µM SC144 inhibited proliferation alone (*p* < 0.001) or in combination with 1 or 5 nM paclitaxel (*p* < 0.001 versus control and versus paclitaxel) in L3.6pl cells (Figure 2c). Although there was no statistical significance, again, an antagonistic effect of 0.1, 0.2 and 1 µM SC144 on 10 nM paclitaxel-induced proliferation inhibition was observed (Figure 2c). The synergy score was -11, underlining the antagonistic action. In ASPC, proliferation was reduced by 1 and 2 µM SC144 alone or in combination with 5 nM paclitaxel (*p* < 0.001 versus control, *p* < 0.01 and *p* < 0.001 versus paclitaxel). Again, paclitaxel did not enhance the effects observed with the single SC144 treatment (Figure 2d).

### 3.3. Raloxifene/Paclitaxel Combination Enhances Apoptosis In Vitro

**Figure 3:**
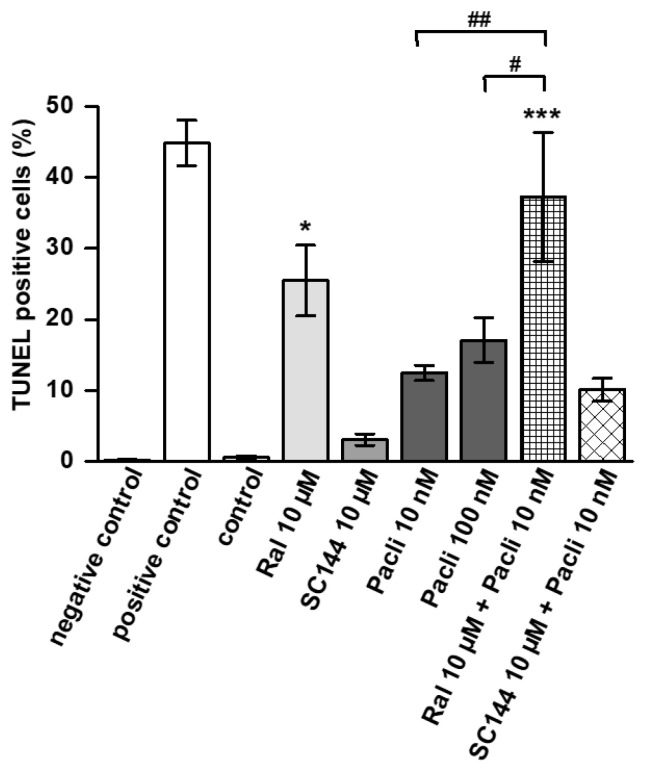
Combined raloxifene/paclitaxel treatment increases apoptosis in L3.6pl cells. TUNEL staining and subsequent FACS analysis were performed on L3.6pl cells treated with different concentrations of raloxifene (Ral), SC144 and paclitaxel (Pacli) alone or in combination. Staurosporine served as positive control, unstained cells served as negative control. The graphs show the mean values ± SEM of n = 4–5. * *p* < 0.05 and *** *p* < 0.001 versus control; ^#^ *p* < 0.05 and ^##^ *p* < 0.01 versus paclitaxel.

Apoptosis induction by paclitaxel, raloxifene and SC144 alone or in combination was assessed by TUNEL staining and subsequent FACS analysis in L3.6pl cells (Figure 3). A total of 10 nM paclitaxel induced apoptosis in 10% of all cells, but this could not be significantly increased by 100 nM paclitaxel. Combined treatment with 10 nM paclitaxel and 10 µM raloxifene increased apoptosis to 37%, and this combination was more effective than 100 nM paclitaxel (*p* < 0.001 versus control; *p* < 0.01 versus 10 nM paclitaxel; *p* < 0.05 versus 100 nM paclitaxel). In addition, 10 µM raloxifene alone was effective in inducing apoptosis (*p* < 0.05 versus control). In contrast, 10 µM SC144 alone had no considerable impact on apoptosis induction, and combined with paclitaxel, it did not intensify the effects of 10 nM paclitaxel (Figure 3).

### 3.4. Combined Therapy of Raloxifene/Paclitaxel or SC144/Paclitaxel Reduces PDAC Tumor Growth In Vivo

**Figure 4:**
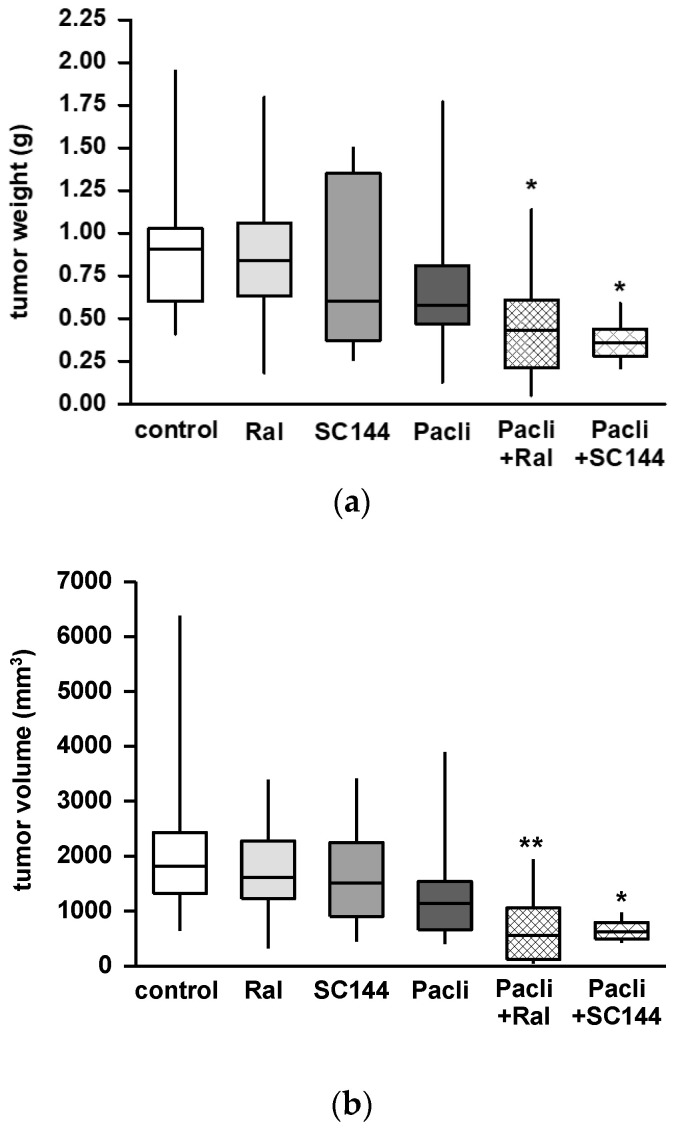
Combination therapy with raloxifene/paclitaxel or SC144/paclitaxel reduced tumor growth in a murine PDAC model. Mice received single or combined therapy with paclitaxel (Pacli), raloxifene (Ral) or SC144 for 26 days after orthotopic tumor cell injection. Combined treatment with raloxifen/paclitaxel (Ral + Pacli) or SC144/paclitaxel (SC144 + Pacli) reduced (**a**) weight and (**b**) volume of primary tumors compared to the control group. The data are given as mean ± SEM of n = 6–17; * *p* < 0.05 and ** *p* < 0. 01 versus control.

Orthotopic injection of human L3.6pl tumor cells into the pancreas of balb c nude mice resulted in tumor growth in 97% of all animals. Two animals died before the end of the experiment. Body weight did not change in any experimental group during the treatment period after surgery. Primary tumors dissected from animals treated with raloxifene, paclitaxel or SC144 alone showed reduced tumor weight (Figure 4a) and volume (Figure 4b). However, this tumor mass reduction did not reach statistical significance when compared to untreated controls. The combination of raloxifene/paclitaxel or SC144/paclitaxel significantly reduced tumor growth and volume in both treatment groups (Figure 4a,b; *p* < 0.05 and *p* < 0.01, respectively, versus control).

In the control group, five animals (29%), and in the SC144 treatment group, four animals (40%) suffered from tumor cachexia. Raloxifene, paclitaxel, paclitaxel/raloxifene or SC144/paclitaxel-treated animals did not show any signs of cachexia. Mice treated with paclitaxel alone had less liver metastasis compared to all other groups (Table 1). Meanwhile, in the control and SC144 treated groups, more than 75% of all animals represented tumor infiltratin of the spleen; this was reduced to 50% in the paclitaxel, raloxifene and raloxifene/paclitaxel treatment groups (Table 1). Peritoneal carcinosis was only observed in the control and in the SC144-treatment group (Table 1).

### 3.5. Combined Therapy of Raloxifene/Paclitaxel Stimulates Apoptosis in Mice’ Tumors

**Figure 5:**
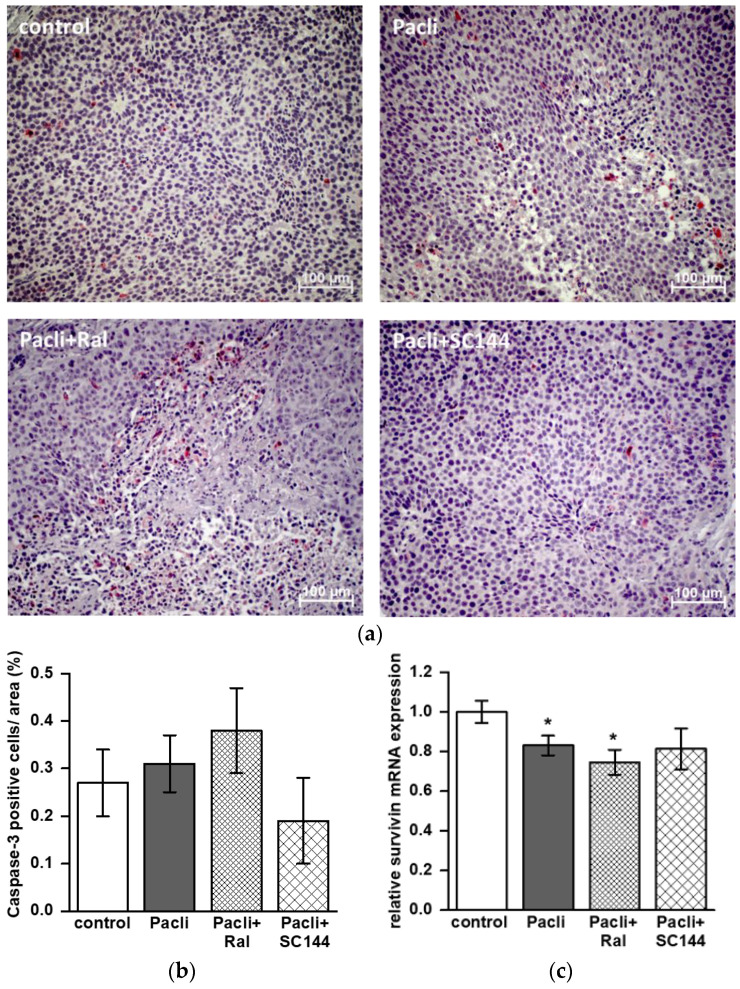
Staining of caspase-3 positive cells of primary mouse tumors and survivin mRNA expression. (**a**) Representative staining of caspase-3 in tumor specimens dissected from control, paclitaxel (Pacli), raloxifene/paclitaxel (Ral + Pacli) and SC144/paclitaxel (SC144 + Pacli) treatment groups. (**b**) Quantification was performed in tumors of n = 5–8 mice of each treatment group. Caspase-3 positive cells were counted in at least five consecutive areas of each specimen (20× magnification), and values are given in percent of positive cells per area ± SEM. (**c**) Relative mRNA expression level of survivin in tumor samples (* *p* < 0.05; Figure 5c).

Tumor tissue dissected from mice treated with raloxifene/paclitaxel showed increased staining of caspase-3 positive cells compared to control, paclitaxel, or SC144/paclitaxel (Figure 5a,b). However, quantification revealed that this tendency failed to reach statistical significance (Figure 5b). For further mechanistic analysis, mRNA was dissected from primary tumors, and the expression of STAT3 target genes was analyzed by qPCR. The mRNA expression of STAT3 itself did not differ in all groups, and a reduction of MMP-7 level in the SC144/paclitaxel treatment group was not significant (supplementary material Figure S3). Paclitaxel alone and in combination with raloxifene (raloxifene/paclitaxel) reduced mRNA expression of anti-apoptotic survivin by about 17% and 25%, respectively (Figure 5c; *p* < 0.05 versus control).

### 3.6. Combined Therapy of SC144/Paclitaxel Reduces IL-6 Expression in Mice’ Tumors and Blood

**Figure 6:**
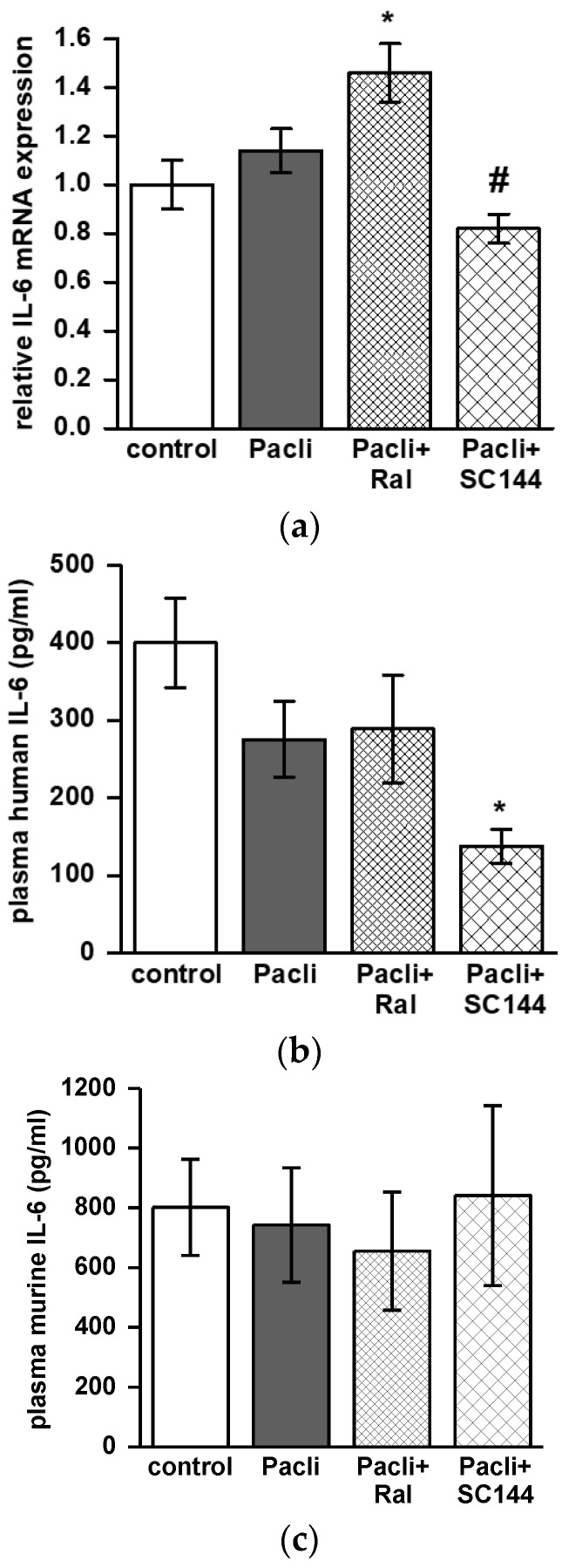
Combined drug treatment with raloxifene/paclitaxel or SC144/paclitaxel changes IL-6 expression in mouse tumors and plasma. (**a**) The graph shows relative mRNA expression level of IL-6 in mouse tumors (n = 4–9). Data obtained from ELISA measurements are given as mean ± SEM and show (**b**) human IL-6 concentration and (**c**) murine IL-6 concentration in mouse plasma (n = 6–12). * *p* < 0.05 versus control; ^#^ *p* < 0.05 versus paclitaxel.

IL-6 mRNA expression was reduced in tumors dissected from mice treated with SC144/paclitaxel compared to single treatment with paclitaxel (*p* < 0.05; Figure 6a). In contrast, raloxifene/paclitaxel treatment enhanced IL-6 mRNA levels compared to untreated controls (*p* < 0.05; Figure 6a). The capability of SC144/paclitaxel to reduce human IL-6 levels was also detected in murine plasma samples. ELISA was conducted and revealed reduced human IL-6 levels in plasma derived from the SC144/paclitaxel treatment group compared to the control group (*p* < 0.05, Figure 6b). An ELISA performed for murine-IL-6 on the same plasma samples revealed no differences between the treatment groups (Figure 6c).

## 4. Discussion

The present study investigated the combined effect of the standard chemotherapeutic paclitaxel with raloxifene or SC144, two molecules interfering with STAT3 signaling by targeting gp130. In vitro analysis showed that raloxifene acts additive to paclitaxel to decrease proliferation and viability and increases apoptosis in PDAC cell lines. In contrast, SC144 acts antagonistic, at least at higher doses of paclitaxel. Combined therapy with either raloxifene/paclitaxel or SC144/paclitaxel reduced tumor growth and tumor volume in a PDAC mouse model. mRNA analysis and immunohistochemical staining suggested raloxifene/paclitaxel stimulated apoptosis in vivo, while SC144/paclitaxel treatment reduced human IL-6 levels in mice tumors and plasma.

Within the last decade, molecular targeting approaches gained increasing attention in oncology. Several studies revealed that IL6/gp130/STAT3 signaling plays a key role in PDAC disease aggravation [9]. Activation of the IL-6/gp-130 leads to a subsequent Jak2/STAT3 phosphorylation [9] which stimulates the expression of IL-6 itself and other pro-survival and anti-apoptotic genes, including survivin [19] or metalloproteinase 7 (MMP-7) [20], which play a role for cell cycle regulation or metastasis, respectively. In previous studies, we showed that raloxifene, as well as SC144, are able to inhibit cell viability and proliferation in PDAC cell lines [11,12]. Therefore, we hypothesized that the efficacy of paclitaxel could be enhanced while its doses might be reduced when combined with raloxifene or SC144.

Our findings on the combined treatment with raloxifene and paclitaxel support this hypothesis, as raloxifene acts synergistic or at least additive to paclitaxel to reduce cell viability and proliferation in vitro. These findings were complemented by our in vivo data. Mice receiving combined therapy of raloxifene/paclitaxel showed reduced tumor growth and metastatic spread to the liver, spleen and peritoneum. Although quantification of caspase-3 positive cells in primary tumors failed statistical significance, there was a clear trend of increased apoptosis in the raloxifene/paclitaxel group. This is supported by our mRNA data that show raloxifene/paclitaxel reducing survivin expression in mouse tumors and by TUNEL staining in vitro showing raloxifene enhancing the apoptotic potential of paclitaxel in L3.6pl cells. Survivin, also known as BIRC5, is highly expressed in most tumors and is associated with poor prognosis in cancer patients, as it promotes cell proliferation and blocks apoptosis [21,22]. Recently, a study proved inhibition of survivin expression by specific inhibitors acts anti-proliferative and induces apoptosis in primary pancreatic cancer lines [23]. In a former study, we observed reduced Ki-67 expression in raloxifene-treated mice in our orthotopic PDAC mouse model, pointing to its anti-proliferative effects [12]. Interestingly, bazedoxifene, which is also a selective estrogen receptor modulator inhibiting gp130/STAT3 signaling, was recently studied in ovarian cancer. Very similar to our data on raloxifene/paclitaxel, combined therapy of bazedoxifene with paclitaxel inhibited cell viability, increased apoptosis in vitro and reduced tumor growth in an ovarian tumor mouse model [24].

Although SC144 alone reduced cell viability and proliferation in PDAC cell lines markedly, it did not act synergistic or additive to paclitaxel. Especially at a higher concentration of 10 nM paclitaxel, the effect turned out to be antagonistic in vitro. Interestingly, in our PDAC mouse model, the combination therapy of SC144 with paclitaxel, but not SC144 monotherapy, was successful in reducing tumor growth. Noteworthy, mice received 1.5 mM paclitaxel every five days, which is a much higher dose than applied in the in vitro assays. This argues against an antagonistic interaction at high doses. From these findings, the type of SC144/paclitaxel interaction on tumor cell viability and growth cannot be sufficiently determined and needs more investigation in ongoing studies.

In contrast to raloxifene, SC144 did not stimulate apoptosis, neither in L3.6pl cells in vitro nor in mouse PDAC tumors. A study on ovarian cancer demonstrated SC144 to induce apoptosis in ovarian cancer cells but not in epithelial cells of the kidney or the endometrium [25]. The same study revealed that SC144 inhibited STAT3 phosphorylation and subsequent expression of survivin, Cyclin-D2 and MMP-7 in a human ovarian cancer cell line [25]. Within the present study, we observed SC144 to inhibit the expression of IL-6 in tumors and plasma. IL-6 plasma levels are typically increased in PDAC patients and are discussed to correlate with metastasis and cachexia [2,26]. IL-6 is produced by the tumor cells themselves or by different tumor-infiltrating immune cells or stromal cells [27,28]. We measured human and mouse IL-6 levels in plasma to figure out if IL-6 was specifically produced by the human L3.6pl tumor cells or mouse immune cells. Interestingly, SC144/paclitaxel combination therapy only reduced human IL-6 levels in plasma, pointing to its direct interference with tumor IL-6 expression. As STAT3 signaling also promotes IL-6 gene expression, inhibition of STAT3 signaling by SC144 could interrupt this autocrine feedback loop [29].

Combined SC144/paclitaxel but not SC144 monotherapy reduced the incidence of peritoneal metastasis and cachexia markedly in our mouse model. However, metastatic spread to the liver and spleen was as high as in the control group. In addition, raloxifene- and raloxifene/paclitaxel-treated animals revealed no cachexia or peritoneal metastasis, although these therapies did not affect IL-6 levels. Presently, the role of IL-6 in cachexia is discussed controversially. While a very recent study reported that cachexia in PDAC is mediated by trans-signaling of tumor-derived IL-6 among tumor, muscle and fat tissue [30], others showed that circulating IL-6 levels correlate rather with advanced disease stage but less with weight loss and cachexia in PDAC patients [31]. Moreover, IL-6 impedes chemotherapy-induced anti-cancer immune responses [32,33]. Doxorubicin treatment inhibited PDAC tumor growth in IL-6 knock-out mice or wild-type mice receiving combination treatment with doxorubicin and IL-6 receptor antibodies [32]. Another study showed that suppression of IL6 derived from a subset of carcinoma-associated fibroblasts reduced PDAC tumor burden in gemcitabine-treated mice. This was associated with an increase of effector T cells in the tumor microenvironment suggesting improved efficacy of immune checkpoint blockade in PDAC [33]. This might also be a mechanism of action of SC144 in vivo and could explain the apparently contradicting findings on SC144/paclitaxel obtained from cell culture experiments and the PDAC mouse model. Because SC144/paclitaxel treatment reduced IL-6 levels in vivo, paclitaxel might act more effectively in reducing tumor size and volume.

## 5. Conclusions

In conclusion, our present study performed on human PDAC cell lines and a PDAC mouse model revealed that raloxifene enhanced paclitaxel efficacy by inhibiting proliferation and inducing apoptosis, while combination therapy of SC144 and paclitaxel improved anti-tumor effects reducing tumor-specific IL-6 expression in vivo. These data support the hypothesis that the addition of IL-6/gp130/STAT3 signaling inhibitors to standard chemotherapeutic agent paclitaxel might be advantageous in treating PDAC, while paclitaxel side-effect profile could be concurrently improved with lower paclitaxel doses.

## Figures and Tables

**Table 1:** Metastatic spread and tumor infiltration in the PDAC mouse model.

Metastasis/Tumor Infiltration (%)	Control	Ral	SC144	Pacli	Pacli + Ral	Pacli + SC144
liver	41	50	60	7	30	33
spleen	76	50	80	50	40	83
peritoneum	41	0	10	0	0	0

## Data Availability

All data that support the findings of this study are available within the article, supplementary material and from the corresponding author.

## Supplementary Materials

The following supporting information can be downloaded at: https://www.mdpi.com/article/10.3390/cancers15020456/s1, Figure S1a: raloxifene dose-response matrix—viability; Figure S1b: raloxifene dose-response matrix—proliferation; Figure S2a: SC144 dose-response matrix—viability; Figure S2b: SC144 dose-response matrix—proliferation; Figure S3a: MMP-7 mRNA expression in mouse tumors; Figure S3b: STAT3 mRNA expression in mouse tumors.
