# Supplementary material for: Targeting Interleukin-6/Glycoprotein-130 Signaling by Raloxifene or SC144 Enhances Paclitaxel Efficacy in Pancreatic Cancer
Source: Cancers (Basel). 2023 Jan 11;15(2):456. doi: 10.3390/cancers15020456 (PMC9856922; doi:10.3390/cancers15020456)
Supplement: Supplementary file 1 [file cancers-15-00456-s001.zip › supplementary materials.pdf]

SUPPLEMENTARY MATERIALS

Targeting interleukin-6/glycoprotein-130 signaling by raloxifene or SC144 enhances paclitaxel efficacy in pancreatic cancer

Nina A. Hering, Emily Günzler, Marco Arndt, Miriam Zibell, Johannes C. Lauscher, Martin E. Kreis, Katharina Beyer, Hendrik Seeliger, Ioannis Pozios

a) **b)**

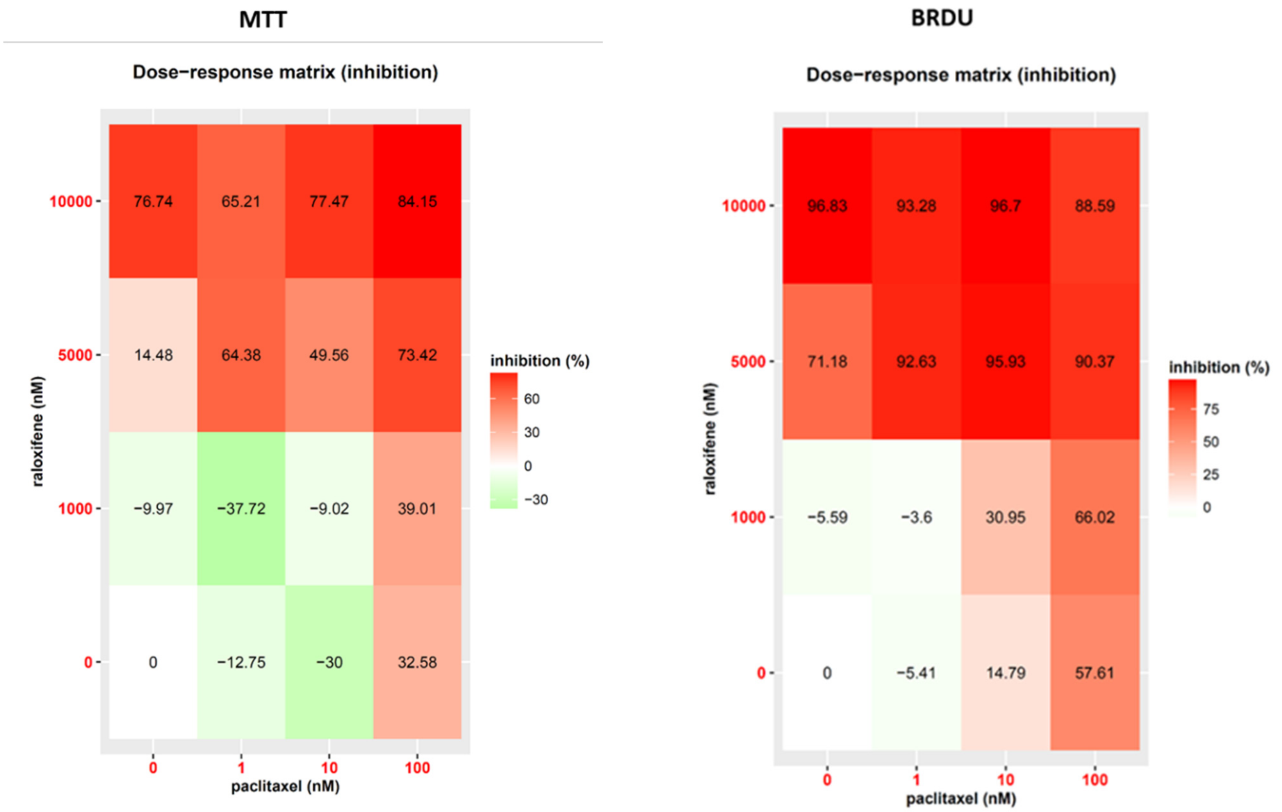

Figure S1. raloxifene dose-response matrix. The dose response matrix shows **(a)** cell viability or **(b)** proliferation inhibition of L3.6pl cells treated with different concentrations of paclitaxel and raloxifene. Data are given in %.

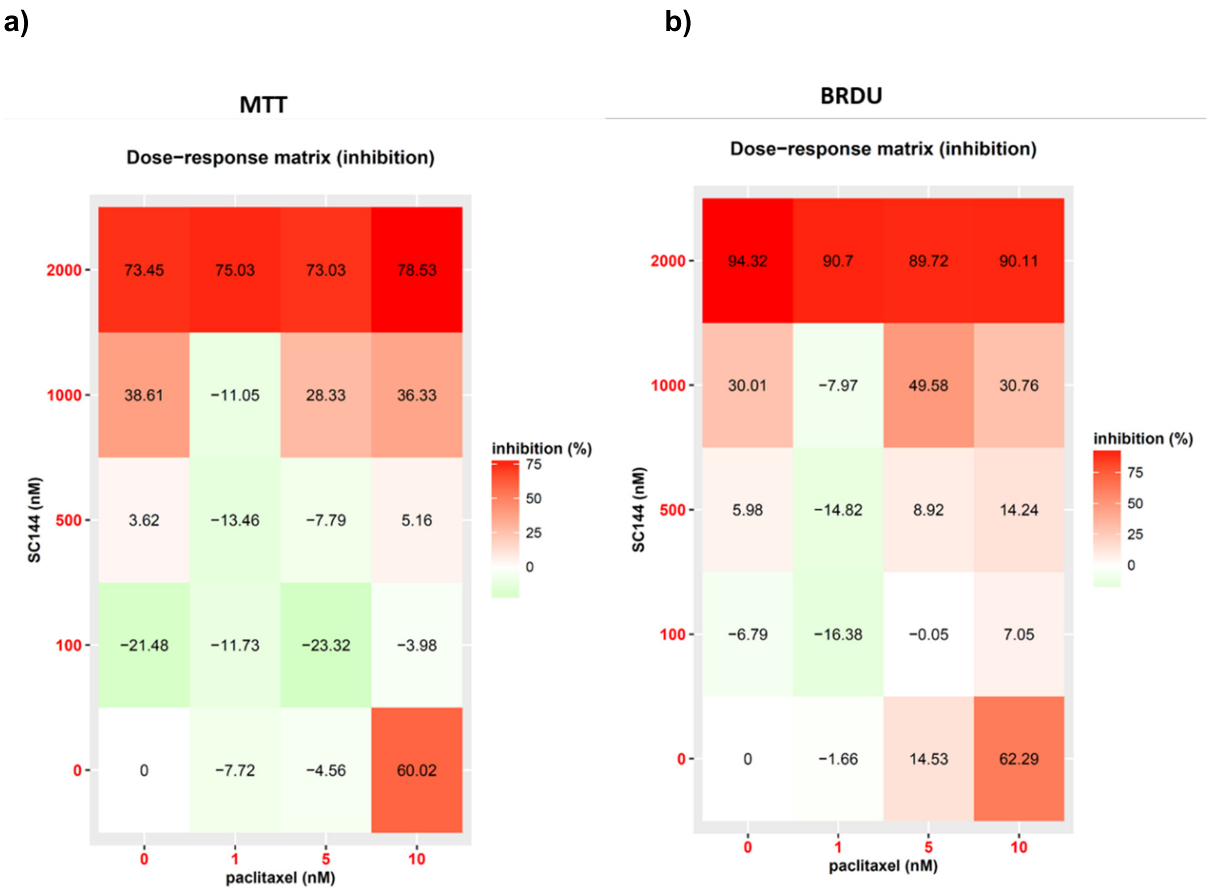

Figure S2. SC144 dose-response matrix. The dose response matrix shows **(a)** cell viability or **(b)** proliferation inhibition of L3.6pl cells treated with different concentrations of paclitaxel and SC144. Data are given in %.

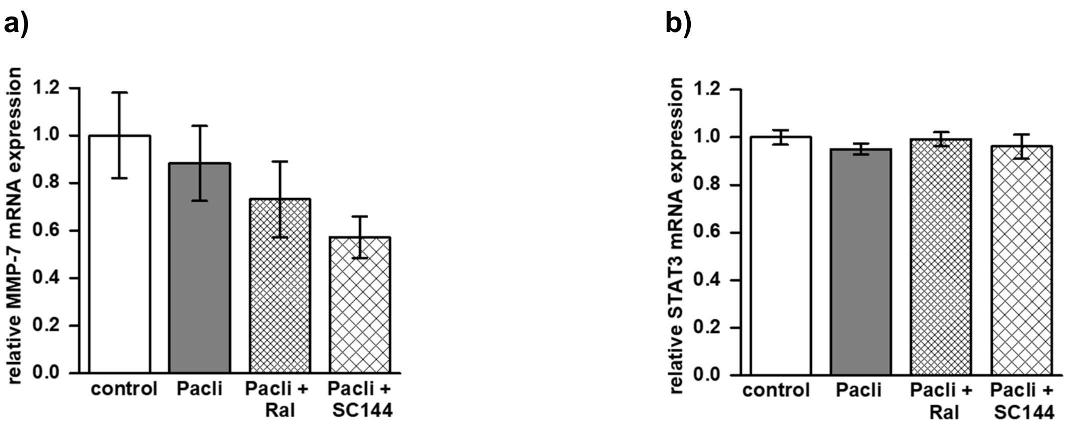

Figure S3. mRNA expression in PDAC mouse tumors of **(a)** matrix metalloproteinase-7 (MMP-7) and **(b)** signal transducer and activator of transcription-3 (STAT3).
